# Encountering suffering in digital care: a qualitative study of providers’ experiences in telemental health care

**DOI:** 10.1186/s12913-023-09367-x

**Published:** 2023-05-01

**Authors:** Jill W. Åhs, Albertine Ranheim, Henrik Eriksson, Monir Mazaheri

**Affiliations:** 1 Department of Health Sciences, Swedish Red Cross University, P.O. Box 1059, Huddinge, 141 21 Sweden; 2 Department of Neurobiology, Care Sciences, and Society, Division of Nursing, Karolinska Institute, Huddinge, Sweden; 3 Section for Health Promotion and Care Sciences, University West, Trollhättan, Sweden; 4 Department of Nursing Science, Sophiahemmet University, Stockholm, Sweden

**Keywords:** Telehealth, Suffering, Patient care, Phenomenology, Lived experience, Mental health providers

## Abstract

**Background:**

Encountering patients who are suffering is common in health care, and particularly when providing mental health care. Telehealth technologies are increasingly used to provide mental health care, yet little is known about the experiences of providers when encountering patients who are suffering within remote care. The present study explored health care providers’ lived experiences of encountering patient suffering during telemental health care.

**Methods:**

A qualitative phenomenological approach was used to uncover participants’ experiences. In-depth interviews were conducted with a purposive sample of physicians, psychologists, and therapists who used telemental health in varied clinical practices in Sweden. Data were analyzed using descriptive phenomenology.

**Results:**

Telehealth care with patients who were suffering was experienced by providers as loose connections, both literally in compromised functioning of the technology and figuratively in a compromised ability connecting emotionally with patients. Providers’ lived experiences were explicated into the following aspects: insecurity in digital practice, inaccessibility of the armamentarium, and conviction in the value of telehealth care. Interpersonal connection between patient and provider is necessary. Worry and guilt arose for providers with fears that technology would not work, patient status was deteriorated, or the care needed could not be delivered. Providers overcame barriers in telehealth encounters, and expressed they perceived that patients appreciated the care received, and through it found relief.

**Conclusions:**

This study brings an understanding of experiences in providing telemental care for patients who are suffering. Providers experience challenges in connecting with patients, and in accessing tools needed to enable reaching the goals of the caring encounter. Efforts to ensure functioning of technology, comfort with its use, and accessibility of tools might be some accommodations to support providers for successful and rewarding telehealth care encounters.

## Background

Encountering patients who are suffering is common during the provision of care [1, 2]. Across specialties and practices, suffering is important to attend to when providing care to help patients heal from ailments and cope with challenges they face, including in mental health care [3, 4]. There are common attributes, but also variation, in how suffering is characterized across medical and health care sciences. It can be seen as severe distress that compromises a person’s sense of wholeness [2]. It is also construed as a loss of meaning in life, the inability to be connected to one’s core life values, or a loss of control or freedom to act and feel engaged in the world with others [5]. Suffering may be attributed to physical or psychological distress, experienced or anticipated [4]. It may also be due to personal or social consequences of illness or disorders, or it may be embodied within social problems encountered in society, such as subjugation, violence, or poverty [2, 6]. Suffering is highly subjective [7] and individual to each patient [8]. Across health care contexts, however, suffering may be what prompts care-seeking [8, 9].

In many clinical practices, use of telehealth is growing ever more common. Telehealth care is increasingly used in mental health care to provide a wide variety of psychological, psychiatric and psychosocial services, also called telemental care. Telemental care has been practiced in Nordic countries since the mid-1990s [10] and such mental health care via video, telephone, internet or other communication technologies has been growing more common in numerous settings globally, even prior to the COVID-19 pandemic [11, 12]. Telemental care has been shown to be effective for use in treating conditions like anxiety [13] and depression [14] and to have broad utility across patient groups and models of care [15].

Telehealth more broadly can play a role in limiting risks like COVID-19 [16] or other exposures for vulnerable patients, and it demonstrates beneficial aspects for patients, healthcare personnel and organizations. For instance, telehealth can increase patients’ access to care, benefit quality and cost-effectiveness of care [17] and studies document patient and provider satisfaction, and even preference, for telehealth [18, 19]. Yet, limitations of telehealth have also long been noted.

Particularly for telemental care, the capacity of the technology itself, such as low bandwidth, has been suspected to limit visual conveying of symptomology, which can be needed for behavioral observations [20]. As a result of constraints within the context of the COVID-19 pandemic, and a rapid switch to remote mental health care, the most impactful concern reported in a survey of therapists was the emotional connection with patients, to express and feel empathy, and read their emotions [21]. Thus, with diminished ability to observe behavioral symptoms [20] or read the emotions of patients [21] due to the characteristics of telehealth technologies, providers might find it difficult to recognize or respond to a complex phenomenon like suffering in telemental health care [22].

Experiences of providers encountering patient suffering in telemental health care has not yet been explored in the literature, despite growing use of this modality [11, 12] and the important role of attending to suffering in mental health care [3, 4]. As such, the aim of the present study is to explore providers’ lived experiences of encountering patients who are suffering in telemental care. This is particularly timely, as telehealth is increasingly practiced, while, at the same time, understanding patients’ suffering is garnering greater attention as a key aspect of care, to help patients effectively cope and heal [8].

## Methods

### Approach

A qualitative phenomenological approach was used in order to uncover participants’ experiences of the phenomenon under study. Phenomenology can be characterized as a structuring of experiences, to further an understanding of the experience, or what it is like to live the experience according to the “experiencer” [23]. This approach suits the study’s aim to garner the implicitly subjective human experiences of health care providers when encountering patients who are suffering in telemental care.

### Setting

The study was conducted with providers of telemental health care practicing in urban, peri-urban and rural areas in Sweden. In Sweden, patients can attend video-based health care meetings on their internet-enabled devices via regional public health systems’ own telehealth applications or those of private providers. Public and private health care services, including mental health and telehealth care, are part of the universal health care scheme, where care is highly subsidized for users. During the period when data collection interviews were conducted, December 2020 through March 2021, health care meetings were often held remotely when possible to limit spread of the SARS-COV-2 novel coronavirus.

### Participants

Clinic heads at more than 80 public and private clinics in Sweden were informed about the study. Managers were emailed an information sheet about the study, and asked to share the information with providers of telehealth care in their clinics. Additionally, some participants suggested their colleagues with rich experience in the domain of the study to be contacted, so they were contacted as well and received the emailed information sheet. This information sheet included a description of the aim of the study, inclusion criteria, and how to take part. Participants were eligible to take part in the study if they had experience providing telehealth care. Interviews were held with seven providers, 3 males and 4 females, ranging in age from 32 to 57 years. Two physicians, one social worker and four psychologists took part. They worked at primary care and specialty clinics, in both the private and public sector. Across their practices, all participants provided some form of mental health care or therapy. They had specializations including neuropsychology, pain rehabilitation, or worked with specific clinical psychiatric populations.

### Data-gathering via In-depth interviews

An open-ended phenomenological approach to in-depth interviews was taken to elicit providers’ detailed descriptions of their lived experiences. The interviews were conducted in an open conversational manner [24] and loosely referenced an interview guide developed by the research team, with warm-up questions about suffering and care to build rapport. The interviewer utilized a primary focus question to lead the conversation to the phenomenon of inquiry: “Could you please describe for me, in as much detail as possible, a digital encounter with a patient who was suffering?” Interviews were interactive and allowed for questioning and probes to guide participants to build on their responses and provide further details or additional experiences to complement what was shared.

Interviews were offered in English, or in Swedish if participants preferred. Of the seven interviews, five were conducted in English and two in Swedish. One interview was conducted face-to-face while the remainder were conducted via video-meeting technology to mitigate risk of virus spread during the COVID-19 pandemic. Interviews were digitally recorded and ranged from 45 to 75 min in duration. The interviewer compiled field notes after the interviews. Recordings of the English language interviews were transcribed verbatim by the lead investigator, and interviews conducted in Swedish were simultaneously transcribed/translated to English by an experienced bilingual research transcriptionist.

### Data analysis

The analysis was based upon the descriptive phenomenological psychological method of Amadeo Giorgi [25]. The authors adopt a psychosocial perspective and seek to inform basic understanding and professional practice. Giorgi’s method was used to explore providers’ experiences, and the roles of psychological concepts, such as emotions and interpersonal relations, coming forth in such experiences [25].

The team of four investigators all had previous experience conducting qualitative research in the health care sciences. In carrying out this analysis, as a first premise, the investigators acted to intentionally suspend preconceived knowledge. The goal of this process is to avoid inadvertently coloring the data or its analysis with ideas to support or dispute any hypotheses relating to the research question. Discussions were held by the research team before and during the data analysis process. The team agreed that embracing of conventional, social or personal presuppositions would be innately counter to the study’s aims. The study focused on exploring lived experiences through meanings shared by those who lived them. The main result of this step is commitment to openness with the approach, to uncover findings grounded in the data and elevate possibilities of the meanings within. The descriptive phenomenological data analysis proceeded over a sequence of steps of (1) familiarizing with all data, (2) delineating meaning units, (3) transforming the units into expressions of meanings lived by the participants and (4) crafting the informed meaning structure [25].

In the initial step, to ensure familiarity with the data, the lead investigator listened to the audio-recorded interviews repeatedly, and carefully read and re-read each transcript, to have a feeling and familiarity of the data within each interview, yet a holistic sense of the body of data as a whole. Meaning units were then delineated as brief statements or lengthy narratives, as necessary, to retain elements of their contextual meaning.

In the next step, relevant meaning units, those expressed by participants in the context of the situation and in relation to the phenomenon being studied, were clustered by patterns and similarities, and then transformed into psychologically informed descriptions. They were then triangulated within the research team, reflecting upon the meaning units and descriptions, resubmerging them within the whole data, dwelling upon and reformulating, and drawing them out further informed. In imagining variations, the characteristics essential to the phenomenon were explicated into expressions to summate lived meanings of the phenomenon. The final phase was to discern the general structure of the phenomenon, the essence, or whole of the meanings of the lived experience, and also present the constituents, or essential parts [26].

### Ethical considerations

This research was carried out in accordance with the principles of the Declaration of Helsinki [27], data and confidentiality was protected, and an informed consent process was upheld. All participants provided written informed consent and agreed to recording of their interviews and use of their data in this research. For interviews that were not conducted face-to-face, participants provided a signed digital copy of the consent form to the research team. Approval for this study was granted by the Swedish Ethical Review Authority (Dnr 2019–06412).

## Findings

### General structure

Telemental health encounters with patients who are suffering is experienced by care providers as a “loose connection” between the provider and patient in the (co-created) telecare encounter. Literal and figurative manifestations of this compromised connection emerge with challenges to both technical and emotional connections in the telemental care encounter. The providers’ lived experiences of remote encounters with patients who are suffering have been explicated into constituent aspects of insecurity in digital practice, inaccessibility of the armamentarium, and value in provision of remote care. Interpersonal connection between the patient and provider is necessary for the provider to engage with the patient successfully and to meet the patient’s clinical needs. Worry emerges, as does guilt, that technology might not work well, or that the patient’s status or situation could be worse than expected, and the provider would not be able to deliver the form of care or level of care needed. Doubt in one’s own abilities, and in the possibility, to provide appropriate care for patients in the telemental care encounter emerges. There is an underlying foundation of the value in the practice and in the provision of telemental care, expressed as a conviction that patients appreciate the care received, and through it find relief. Further description of the constituent aspects proceeds below.

### Insecurity in digital practice

#### “Insecurity” in the digital medium

Care providers meet challenges in the telemental health care encounter that impact their ability to diagnose conditions, and ability to respond to a patient’s condition or situation. Experiences of insecurity, anxiety, worry, and guilt, came forth in providers’ encounters with suffering, arising as doubt in one’s own abilities and judgment, and tied to complications arising from the technology used. Providers worry about the telecare technology not working, which can prevent or delay the start of the telemental care appointment, or abruptly and unintentionally end a patient encounter. Worry precedes telehealth encounters for providers as they face this *“nervousness” P3* and “…*anxiety; will the tech work, or not?” P5*.

Worry and insecurity arise also in not knowing what will be met in the care encounter, such as diminished mental status of the patient, or unknown severity of their situation, or not knowing the patient well. This results in the mental health care provider being apprehensive and acting differently from non-digital encounters. The provider might not be at ease when providing telemental care, as they might be insecure in interpreting the status of the patient, or the situation or environment the patient is in. The providers “*do not know what to do in that conversation*” *P3* where they encounter the unexpected, or a patient becomes very upset. The provider might instead then be tiptoeing in the telehealth conversation, unsure how the patient will react: “*I am more careful.” P3*.

It is challenging not only to meet such a patient, but also to respond to a patient in need, particularly if the patient’s status is worse than expected. The ability to convey understanding and craft a sensitive and appropriate response seems to be lost through the medium. *“It is so much harder to face. It is harder to be reactive in an adequate way.” P3* Insecurity emerged as doubt and compromised confidence in the ability to diagnose or recognize needs of their patients, and thus worry about the ability and possibility to respond appropriately.

Providers may be confused or wonder what is truly happening with the patient during the digital encounter. One encounter described as suspected dissociation in a patient with history of trauma who “*looked up and down and around*”*P3* was actually due to distraction by a highly active cat in the client’s home. Insecurity in what is seen or encountered in the telemental care session extends into the act of documenting diagnoses or patient status in the medical charts: *“… like, I want to ask, ‘Are you crying now?’, it can be because people are in darker rooms. It can be hard to see and interpret the situation. … it shows when you are to write a psychiatric status in the journal, that there is an insecurity in that: What was it that I actually saw?*” *P3*.

#### Insecurity in the (emotional) connection

A compromised sense of interpersonal connection with the patient arises in the mediating features of the technology “…*when you sit through a link… it is hard to get that connection, that, yeah, the deeper connection in a way.” P3* The telehealth medium hinders the provider from feeling a close interpersonal connection with the patient. The symptoms of a disorder, such as problems with social functioning, may impact a patient’s ability to connect with others. This might be more apparent when communications are through digital media. “*[W]hen you do not get a connection, [it] is a greater suffering*.” *P3* This is experienced as feeling a lack of emotional closeness, or a weaker connection with the patient in the digital encounter.

Arranging an in-person meeting with the patient first “*to create a connection*…”*P3* is beneficial as “*it happens something there… if you meet them first, … get to know you, a little bit of face to face… I would say the follow through in the therapy is a little bit better.*” *P2* Providers value meeting with the patient in person for a first encounter, feeling that they can get to know the patient better this way and establish a connection.

### Inaccessibility of the armamentarium

#### Unable to respond as one would like

In the digital meeting, providers are held to deliver care without access to their full arsenal of tools. They are not able to use the key approaches they relied on previously, those which they have been trained in, and well-practiced in. Providers are at a disadvantage when they no longer have their tried-and-true tools and techniques at their disposal, particularly when responding to suffering in the digital care encounter, they might “*… feel indigent in my arsenal of how I can deal with it”. P4*.

When meeting a patient face-to-face in the clinical setting, providers offer comfort through actions like to *“take a glass of water” P5* or “*putting some tissues on the table” P3*, or through gestures like “*having a relaxed body language” P3*. In the digital meeting, though, efforts to indicate sympathy instead feel “…*forced. Strange.” P5* Sharing the experience with the patient of being together in the office space, or sharing the routine of closing of the session by walking together with the patient to the door, are comforting acts lost in the digital meeting. Techniques the provider might rely on, such as taking a long pause to allow time for the patient to reflect, is instead avoided, so as not to provoke angst in the digital meeting if interpreted by the patient as a technical problem.

In the physical space of the clinical office, when patient and provider are meeting in-real-life, there is something shared in a patient-provider connection of being present together. In the digital meeting, the provider has little more than what they can express in words, which can come across more crude. *“On video, the feeling is that you need to validate only by the spoken word, what you say becomes the most important and you lose the nuances of how I say things.” P3* Maybe the provider would speak softly to the patient in the clinic or would explain something in a gentle way. But, the intention for speaking a calm tone is lost in the telehealth medium, particularly if the provider must clearly articulate a message, such as next steps to ensure the patient’s comprehension or their safety. Not only do the words spoken have more weight, but the way in which they are expressed is more strained, and any delicacy might be lost. What is said, and how, becomes amplified in importance.

#### Undermined therapeutic atmosphere

In telemental care, providers experience a loss of power and control, compared to in-person encounters. By the very action of the patient showing up to the clinic, the provider sees a demonstration of commitment to care.

*“If they have come here and sat down in my chair, …that’s already a win, somehow. I know that I have them on my side. … At home and over digital media… it’s easier to just pick up your phone and answer a call… They don’t have to be as motivated to do that. So if they’re here, …somehow… I have the upper hand already.” P4*.

In telemental care encounters, the provider experiences missing the “edge” of having the patient in their physical environment. The provider does not have comfort in knowing the situation and feeling in control of handling it. For example, it was important to providers to be able to respond to a patient having an emergency or in a potentially dangerous situation. “*It helps a lot to feel as a clinician more that the situation is safe.” P6*.

### Importance of meaningfulness of care provided

#### Telemental care is a valued service to provide

The care providers experience being valued and being of help to patients through the very acts of talking and of engaging with them. This is despite being challenged by lack of preparation or technical problems with equipment. *“People know that you try to help them, in any case. And we have found this way, [with] video… Yeah, … got [a] problem with the microphone, with [the] camera, but I’m… I’m here. …And this is something that helps patients.” P5*.

Providers recounted that, even when technology is not working and there is difficulty with the logistics of the digital care, the provider can feel the care is of benefit to the patient. The sharing and relation between them helps to relieve the patient’s suffering. And even *“If they can’t get a time, an appointment in two days, because … we are full of requests … this is something that can help sometimes, ’OK, I’m not alone’.” P5*.

#### Keeping it meaningful for providers can be a struggle

The provision of digital care *“is less satisfactory work*” *P3* for the provider than in-person care. This occurs in light of experiencing insecurity, for example, as *“If I have had a person in the room, I can feel that I trust my assessment. …But after I videocall… It is harder to trust your own assessment. It is more a feeling of being insecure afterwards and less satisfaction.” P3*.

If there is a technical difficulty that prevents or interrupts the encounter, no matter if it is on the patient’s side or the provider’s, the provider finds “*It’s always my… It’s always my problem somehow.* P4” The provider bears the responsibility and guilt, no matter the reason for technological problems. *“That’s the responsibility part of it. So even if it’s… If it’s not me, I feel like… I could have maybe done something anyway.”* P4.

Particularly in light of guilt, or insecurity, or feeling like you do a worse job, the work is less rewarding and “*everyone agrees that our work is less interesting and meaningful now and that is really, really sad that we don’t get to meet persons, like in real life.” P3* Providers experience a challenge in keeping digital care as meaningful as their on-site caring encounters.

## Discussion

This study describes experiences of telemental health care providers who encounter patient suffering in digital care. The essence of the phenomenon is summated as “loose connections” that can compromise attaining the therapeutic goals of the caring encounter and a distance between patient and provider in telemental care encounters. Characteristics of the digital medium in telehealth can result in challenges to feeling a connection and a psychological distance co-created by the patient and provider. This can impact shared expectations and experiences in this (physically) distant form of care. Providers’ experiences were tethered to their commitment to care, described through value placed on connecting with their patients and meeting patients’ clinical needs.

The “distance” co-created between patient and provider in the telemental care encounter can be construed in a number of ways [28] not least as spanning across literal space, or the physical distance between patient and provider, but also framed in a compromised connection, in both a technical sense and also impacting the emotional or interpersonal connection between patient and provider. Literal manifestations of “loose connection” arose in the present study in technical problems causing delays, interrupting video or sound, or which may literally and suddenly jeopardize the digital connection, and results in a feeling of insecurity or fragility of a potentially fleeting caring encounter. Providers experienced worry about sudden and unpredictable technical failures, and negative impacts due to technology compromising success of the care encounter.

Providers equated the technology with limits to communication with patients, similar to previous research pointing to mediating roles of technology in barring true eye-contact [29] or impairing observation [29]. In this way, a figurative “loose connection” emotionally between patient and provider can be attributable to the nature of the digital medium, where some interactions are not possible or are obstructed. Technology has an ever-important role in enabling or impeding digitally-mediated care. It may negatively impact both verbal and non-verbal communications, including sounds and physical gestures [30]]. In the present study, the inability to offer comfort through gestures or body language was problematic, and experienced as impossible due the practicalities of telehealth technology. This can impair the rapport-building providers may otherwise be accustomed to, and which may be considered imperative for the therapeutic relationship [29].

Providers in the present study experienced the sense of disarmament in not being able to access an arsenal of tools to respond to patients’ clinical needs, or offer actions of comfort, such as putting a box of tissues near a patient who was upset. Previous work has described similar challenges for providers in telehealth, such as not being able to offer physical comfort to a patient, like a pat on the arm [31].

Providers were concerned for being able to truly understand patients’ status and being able to accurately diagnose or note these in the medical journal. Similarly, in tele-dermatology, what may have previously been considered a remarkable achievement, to use visible data to make an image-only diagnosis, resulted rather in a “blindness” to the whole patient and the scenarios and problems they face. A narrower or more limited amount of information gathered in remote care and used to inform a diagnosis suggests the risk for misdiagnosis could be greater [28]. Taking into consideration the “whole person” and aspects gleaned from observation of symptoms and behaviors is indispensable when making a diagnosis in mental health care [32].

Protections to ensure a patient’s safety or in case of emergency was a concern for providers in the present study. Though, in the Swedish context where the study was conducted, emergency medical and social service home visits can be arranged in acute situations. However, providers might not be trained how to manage crises outside the clinic. Providers might not know how to inform emergency services to respond when they themselves do not know the patient’s location. They might not be trained how to handle when a patient abruptly ends a meeting. These examples may occur in telehealth, but not in the same way in an office-based encounter [30]. Critically, previous studies indicate that having confidence in practice is essential for the provision of healthcare [33, 34]. Further training or learning through experience may allow providers to develop this necessary confidence in care [35, 36] and particularly to respond within emergency situations.

Finally, providers in the present study candidly expressed that telehealth encounters were less engaging, which is aligned with previous findings that virtual meetings are perceived to be “not as fulfilling” as meeting patients in-person [29] or are more “exhausting” [37]. Work engagement has been found to be an essential part of well-being and negatively predicted depressive symptoms in a prospective longitudinal study among dentists [38]. Similarly, work engagement has been found to be associated with job satisfaction, intention to stay in the profession, and quality of care among nurses in psychiatric hospitals [39]. This may be an important consideration for providers and health services managers. There may be potential negative implications for practice as well as negative health consequences for providers from the impacts of experiences like those described in this study. Offering opportunity for providers to express their experiences using this modality and to request any needed support or training to improve their telemental health care could be helpful. Creating forums where providers can share their learnings as well as strategies used to overcome limitations of telehealth modalities could also be fruitful. More research on this topic may help understanding how negative impacts could be avoided or ameliorated at the individual and organizational levels.

### Methodological considerations

Interviews were conducted in English or Swedish, and for participants whose mother-tongue was neither language, this might have impacted their abilities to freely describe their experiences.

Also, findings are from care providers of a diversity of experiences yet may vary from experiences of other healthcare providers. This study does not purport to uncover generalizable findings, but rather to illustrate expressions of life-world experiences relevant to the phenomenon of inquiry. Additional research with other participants may offer further valuable or different insights.

## Conclusions

This study brings an understanding of providers’ experiences encountering the suffering of patients within telemental care. Providers experience distance in connecting with patients, and in accessing tools needed to reach the goals of the caring encounter. In telemental care, a “loose connection” emotionally may be accompanied or exacerbated by a literal “loose connection” in the unreliability of the digital technology. Limiting characteristics of the telehealth medium result in experiences of insecurity for providers to confidently meet patients’ clinical needs. Providers find value in offering telemental care for patients, and efforts to ensure functioning of technology, comfort with its use, and accessibility of tools might be some accommodations to support providers for successful and rewarding telemental care encounters.

## Data Availability

Requests may be addressed to the corresponding author. In upholding informed consent and protecting confidentiality, data cannot be shared nor made publicly available.

## Declarations

### Guidelines Statement

All methods were carried out in accordance with relevant guidelines and regulations.
